# Fever treatment in the absence of malaria transmission in an urban informal settlement in Nairobi, Kenya

**DOI:** 10.1186/1475-2875-8-160

**Published:** 2009-07-15

**Authors:** Yazoume Ye, Nyovani Madise, Robert Ndugwa, Sam Ochola, Robert W Snow

**Affiliations:** 1 African Population and Health Research Centre, Nairobi, Kenya; 2 School of Social Sciences, University of Southampton, Highfield Southampton, UK (Affiliated to African Population and Health Research Center at the time of conceptualizing this project); 3 Centre for Population Studies, London School of Hygiene and Tropical Medicine, London, UK; 4 Provincial Medical Officer for Health, Nairobi, Ministry of Health, Kenya; 5 Malaria Public Health and Epidemiology Group, Centre for Geographic Medicine, Kenya Medical Research Institute-Wellcome Trust Collaborative Programme, Nairobi, Kenya; 6 Centre for Tropical Medicine, Nuffield Department of Clinical Medicine, University of Oxford, CCVTM, Oxford, UK

## Abstract

**Background:**

In sub-Saharan Africa, knowledge of malaria transmission across rapidly proliferating urban centres and recommendations for its prevention or management remain poorly defined. This paper presents the results of an investigation into infection prevalence and treatment of recent febrile events among a slum population in Nairobi, Kenya.

**Methods:**

In July 2008, a community-based malaria parasite prevalence survey was conducted in Korogocho slum, which forms part of the Nairobi Urban Health and Demographic Surveillance system. Interviewers visited 1,069 participants at home and collected data on reported fevers experienced over the preceding 14 days and details on the treatment of these episodes. Each participant was tested for malaria parasite presence with Rapid Diagnostic Test (RDT) and microscopy. Descriptive analyses were performed to assess the period prevalence of reported fever episodes and treatment behaviour.

**Results:**

Of the 1,069 participants visited, 983 (92%) consented to be tested. Three were positive for *Plasmodium falciparum *using RDT; however, all were confirmed negative on microscopy. Microscopic examination of all 953 readable slides showed zero prevalence. Overall, from the 1,004 participants who have data on fever, 170 fever episodes were reported giving a relatively high period prevalence (16.9%, 95% CI:13.9%–20.5%) and higher among children below five years (20.1%, 95%CI:13.8%–27.8%). Of the fever episodes with treatment information 54.3% (95%CI:46.3%–62.2%) were treated as malaria using mainly sulphadoxine-pyrimethamine or amodiaquine, including those managed at a formal health facility. Only four episodes were managed using the nationally recommended first-line treatment, artemether-lumefantrine.

**Conclusion:**

The study could not demonstrate any evidence of malaria in Korogocho, a slum in the centre of Nairobi. Fever was a common complaint and often treated as malaria with anti-malarial drugs. Strategies, including testing for malaria parasites to reduce the inappropriate exposure of poor communities to expensive anti-malarial drugs provided by clinical services and drug vendors, should be a priority for district planners.

## Background

Most studies of urban malaria transmission in sub-Saharan Africa (SSA) suggest significant reductions in the risk of infection compared to neighbouring rural areas [1-7]. These reduced risks of transmission are, in part, related to reductions in suitable habitats for dominant vectors [3,8]. However, localized transmission has been associated with urban agriculture [9,10] and standing permanent water [11]. Knowledge of transmission across the rapidly proliferating urban centres in Africa and recommendations for prevention or disease management remain poorly defined [5,12-15]. The paper presents the results of investigations of malaria infection prevalence, use of preventative measures and the treatment of recent febrile events among a population living in an informal settlement in Nairobi, Kenya.

## Methods

### Study area

Nairobi is a classic example of a rapidly growing human settlement. In 1900, Nairobi was a settlement restricted to a small area between the Ngong, Mathari and Nairobi rivers and expanded quickly with the building of a railway to become the colonial government headquarters in 1905. In 1910, the population of Nairobi was estimated to be only 1,700 people [16]. By 1944, Nairobi had reached a total population of 100,000 and was assigned a "city" status in 1950, and by 1964 had reached a total population of approximately 0.3 million with a population density of 544 people per square kilometre. Between 1961 and 1999 Nairobi's population grew to an estimated 2.14 million with a population density of 3,060 people per square kilometre [17]. This rate of growth has led to the establishment of informal settlements providing mixtures of semi-permanent and make-shift accommodation for in-migrants seeking employment. Between 1971 and 1995, the number of informal, "slum" settlements within the boundaries of greater Nairobi rose from 50 to 134, and the estimated population of these settlements increased from 167,000 to 1.9 million inhabitants [18].

Nairobi was historically an area of seasonal malaria transmission with as many as 939 clinical cases documented each year between 1930 and 1939 [19] and epidemics were documented in 1926, 1935 and 1940 [20,21,19]. In 1912, *Anopheles gambiae *and *Anopheles funestus *were reported as present within the city [22] and confirmed as vectors of malaria in 1926 [23]. *Plasmodium falciparum *infection prevalence among children was reported to be 2.5% in 1929 [19] and 25% in 1946 [24]. Following World War II, there was a decline in reported malaria cases within the city to an average of 261 per year, coinciding with rapid population growth [19,3]. A forty year period elapsed before a systematic survey of malaria infection was reported again. In 1982, *P. falciparum *infection prevalence was reported to be between 1.8% and 13.5% at nine schools across Nairobi [25]. Despite the low prevalence of infection, in 2001 malaria was the second highest disease diagnosed among attendees to outpatient clinics after respiratory tract infections and represented 10.6% of all diagnoses in Nairobi [26].

**Figure 1:**
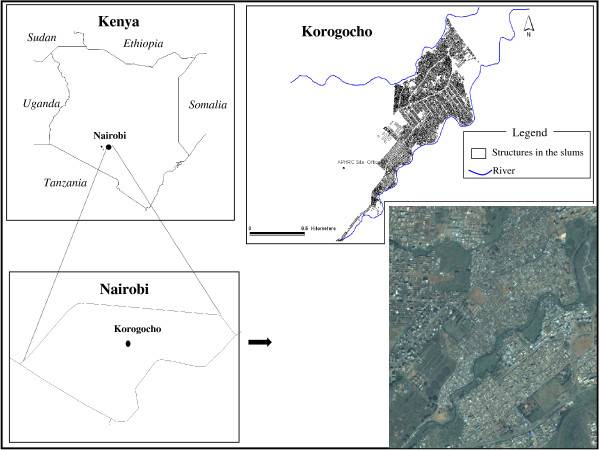
**Study area: location of Nairobi in Kenya (top left panel); location of Korogocho in Nairobi (bottom left panel); distribution of households in Korogocho (top right panel) and Google Earth image of Korogocho slum (accessed January 2009)**.

The present study was undertaken at Korogocho, a slum settlement that forms part of an extended Nairobi Urban Health and Demographic Surveillance System (NUHDSS) established in 2002 to prospectively monitor vital, social and health events [27]. Korogocho is characterized by poor environmental sanitation, low coverage of toilets, absence of sewers or drainage systems and limited access to safe drinking water [28,29]. The slum is occupied by 26,600 people living in 8,800 households on an area of less than 0.6 square kilometres. Korogocho slum is located between Gitathuru River in the north and the Nairobi River in the south (Figure 1). Most houses are made of mud and timber with roofing of waste tin cans. Because of the poor drainage system there are stagnant water pools between the dwellings. The slum is served by 15 formal, public health facilities including two government run health centres, seven facilities run by faith-based organizations or non-governmental organizations and six clinics supported directly by community funds. These services are augmented with an estimated 96 private, for-profit health care providers and 128 retail outlets selling over-the-counter medicines including anti-malarials.

### Study population

The NUHDSS population census is routinely updated and the present study used the *de jure *residents at 1^st ^January 2008 as the sampling frame. All residents of the Korogocho NUHDSS on this date were eligible to be sampled and included in the study if they consented. Using simple random sampling 1,069 individuals were selected. Fieldworkers visited households of the sampled individuals to seek their consent to participate in a cross-sectional malaria parasite prevalence survey.

### Data collection

Four teams of trained interviewers, each comprising of a laboratory technician and a nurse collected the data on individuals who consented to participate in the study. Since the survey involved an oral interview and finger prick blood sampling, individuals could consent to be interviewed but refuse to provide a blood sample. Individuals who consented to participate were interviewed about the usual and previous night use of mosquito nets and whether these were treated with insecticide. Participants were also asked about fever symptoms experienced over the last 14 days preceding the visit and details on the treatment of these episodes. This information was asked for every reported fever episode and included how the fever was detected, the duration of the fever, the type (including formulation) of medicine taken and the origin of the medicine.

After the interview, participants who consented to be tested were finger pricked and a rapid diagnostic test (RDT: Acu-Check^®^, Mannheim, Germany) performed. The RDTs were read 10 minutes after preparation and a confirmation reading was done 20 minutes later. The interviewers also prepared thick and thin slides and labelled them with the participant's identification number. Participants were provided with the results of the RDT and those positive were given treatment with Coartem^® ^(artemeter-lumefantrine), the recommended first-line treatment for malaria in Kenya [30].

Slides prepared in the field were transported the same day to the site office for staining with 3% Giemsa. Two hundred high power magnification fields were examined per slide by two independent laboratory technicians with experience in reading malaria slides. Where readings were discrepant a third reading was used as arbitration. In addition all RDT positive results were re-read again by three independent technicians for parasitological confirmation.

### Data processing and statistical analysis

Data were entered in a database designed in SQL Server 2000 [31] and analyzed using Stata version 9.1 [32]. Descriptive analysis included point and period prevalence (the reference period was two weeks preceding the interview) computed with 95% confidence intervals stratified by age and sex. The proportion of fever cases treated by origin of the medicine, type of anti-malarial medicine, and lag between onset of the fever and beginning of treatment, for different age groups was computed.

### Ethical approval

The proposal was approved by the KEMRI/National Ethical Review Committee (SSC number 1227).

## Results

### Study participants

Of the 1,069 participants 111 (10.4%) individuals reported to have travelled out of Nairobi in the eight weeks preceding the interview. Of the 1,069 residents interviewed, 87 (8.9%) refused to provide a finger prick blood sample. 52% of the 1,069 interviewees were female and 63% were aged 15 years or older.

### Malaria infection and bed net use

Three (0.3%) participants were reported to have a positive *P. falciparum *RDT result at the time of interview. After six separate examinations of 200 high power magnification fields of the thick smears of the three RDT positives, all were defined as microscopically negative. Among all the slides taken during the survey 12 (1.2%) were damaged before being read and an additional 18 (1.9%) were poorly stained and unable to be read. Of the 953 remaining readable slides all were reported as negative for the presence of sexual or asexual forms of *P. falciparum*, *Plasmodium malariae *and *Plasmodium ovale*.

Of the 1,069 participants 110 (10.3%) reported owning a bed net and 103 (9.6%) sharing with another member of the household. The majority (55.8%) of the bed nets owned by study participants were treated with insecticide; of which 38% were pre-treated and 17.8% treated by the owner. Among participants who reported to have access to a bed net (owning and sharing), 88.7% slept under the net the night preceding the survey.

### Period prevalence of self-reported fever episodes

Information on self-reported fever was missing for 31 (0.3%) individuals. Of the remainder, 34 (0.3%) said that they did not know whether they had had a fever or not and, therefore, analysis was restricted to 1,004 interviewees. Overall 170 (16.9%, 95% CI: 13.9%–20.5%) fever episodes in the last 14 days were reported among 136 people. Thirty people reported more than one febrile event and four people reported more than two febrile events. The highest reported fever rate was reported for children less than five years by their parents or guardians (20.1%, 95% CI: 13.8%–27.8%). Self-reported fever prevalence was lower among age groups 5–9 years (6.9%, 95%CI: 3.2%–12.6%) and 10–14 year (8.9%, 95% CI: 4.4%–15.8%) however, period prevalence increased to 19.8% (95% CI: 16.7%–23.1%) among participants aged 15 years or older (Table 1). There were no differences between males and females in any age group.

**Table 1:** Self-reported fever episodes in the past 14 days preceding the surveys

**Characteristics**	Population*	**Episodes**	**Period prevalence (%) [95% CI]**
	**1,004**	**170**	**16.9 [14.7–19.4]**

**Gender**			
Female	524	89	17 [13.9–20.5]
Male	480	81	16.9 [13.6–20.5]

**Age group (Years)**			
0–4	139	28	20.1 [13.8–27.8]
5–9	131	9	6.9 [3.2–12.6]
10–14	112	10	8.9 [4.4–15.8]
15 +	622	123	19.8 [16.7–23.1]

* The total population used is 1,004 instead of 1,069 because information on fever was missing for 31 participants and 34 said they don't know

### Diagnosis and treatment of fever

Participants reported that fevers were diagnosed mostly by a feeling of the body being hot (71.8%) but some of the cases were diagnosed in health facilities (27.1%). 1.2% of the fever episodes were diagnosed at home using a thermometer.

Among the 170 febrile episodes reported, 8 (4.7%) did not have information on treatment. Of the 162 febrile episodes with information of treatment, no action was taken to manage the event for 74 (45.7%). Among the 88 fevers for which an action was taken 85 reported using a modern medicine and three used herbs obtained from traditional healer (Additional file 1), 34.1% of these medicine events occurred on the same or next day from the onset of symptoms and 52.3% after three or more days (Additional file 1). 44.3% of treated fevers were managed at formal public sector health facilities and 17% of fevers paid for medicines at private clinics/pharmacists. 28.4% of fevers were treated using medicines purchased from one of the estimated 128 retail outlets selling over-the-counter drugs within the slum area. Patients older than 15 years were more likely to seek medicines from the retail sector (47.7%) compared to only 16.7% of fevers in children aged less than five years (Additional file 1).

Of the medicines prescribed or self-purchased 32 (36.4%) of interviewees could not adequately describe the medicines they used nor did they have any packaging remaining for interviewers to ascribe the formulation to either an anti-malarial preparation or other oral medications. Among the 56 febrile events for which a medicine type could be established 31 (55.4%) were identified as branded forms of sulphadoxine-pyrimethamine (SP) and 16 (28.6%) were classified as a formulation of amodiaquine (AQ). Interestingly one fever was treated with chloroquine, an anti-malarial drug discontinued as recommended therapy eight years earlier, and one fever was treated with oral quinine, a drug reserved for second-line therapy. Only four fevers, all among adults, were managed using the nationally recommended first-line treatment for malaria, Coartem^® ^or artemether-lumefantrine (AL). Of the identified anti-malarial drugs used to manage 53 fevers 16 were sourced from the informal retail sector as over-the-counter medicines, 12 were prescribed and purchased from a private clinic or pharmacy and 23 were prescribed at the formal, public sector. Within the public sector the tendency was to prescribe SP or AQ and in only four of consultations within this sector and for which the respondent could remember or show evidence of drug-type did the febrile patient take AL.

## Discussion

There remains some controversy over the likelihood and extent of authochronus malaria transmission within Nairobi city [33,34]. Nairobi has grown into a massive urban sprawl in the last few decades with intense population growth in informal settlements. This study of an urban slum community was unable to show any evidence of peripheral infection with malaria parasite among 953 residents. The study took place after the rains in July 2008 and at a time when ambient temperatures remained suitable for sporogony to complete in either *An. gambiae s.s*, *An. funestus *or *Anopheles arabiensis*. The results do not preclude the possibilities of local malaria transmission in this area, but strongly suggest that should transmission occur it would be at exceptionally low levels, representing very rare events. Furthermore, the findings should not be interpreted as excluding the possibilities of malaria transmission in other parts of Nairobi with different settled population densities and surrounding environments, a wider series of investigations would be required to characterize malaria risks across Nairobi's complex and vast limits. There is also a need for an entomological study to characterize the mosquito population and assess the sporozoite rate to able to rule out any local transmission.

Importantly, however, the observation that there were no individuals who were harbouring infection does provide a context to understand the appropriateness of fever case-management practices in this community. Fever was a relatively common complaint in two-week recall period among residents of Korogocho, 12.7% of the sampled population complained of having had a fever and 170 febrile events were recorded among 136 people. Consistent with other fever surveys across Africa [5,6] many febrile events are not managed using medicines or any specific interventions and one assumes these fevers are self-limiting and transient. However actions were taken to manage 88 (51.8%) fevers among Korogocho residents, 85 (96.6%) were managed with some form of pharmaceutical product of which we were able to document through interview and observation and found that 53 (62.3%) were treated with an anti-malarial preparation. Assuming an equivalent drug-exposure history across every two-week period in a year this would represent a minimum estimate of 1.3 anti-malarial drug exposures per person every year in an area where we were unable to detect any local malaria transmission.

Only four of the anti-malarial treatments that could be identified were AL, the nationally recommended first-line treatment, and the majority were either mono-therapies drugs not recommended in the national treatment guidelines (AQ) or therapies abandoned because of wide-spread resistance (SP and chloroquine). Regardless of appropriate treatment regimens the combined findings of zero parasite prevalence and high anti-malarial use suggests that community and prescriber/dispenser education on the need to establish the diagnosis using parasitological detection is critical in this urban setting. These pleas have been made to support fever case management in other urban settings [5,6,35]) and areas of low transmission in East Africa [36,37]. The transition from cheap, readily available mono-therapies to more expensive, restricted access artemisinin-based combination therapy (ACT) in Kenya in 2006 [38] should serve as a further financial incentive to minimize over-diagnosis and inappropriate drug management through applied diagnostics [39,40].

Should diagnosis based on systematic testing for malaria parasites be promoted as a strategy in Nairobi to reduce inappropriate malaria treatment it is important to extend its implementation and support to sectors beyond the formal public sector. Over 52.8% of all fevers treated with a documented anti-malarial in the present study used medicines obtained from the private and retail sectors. These prolific and hard to regulate service providers must be engaged in strategies to reduce anti-malarial drug use in areas where malaria transmission is negligible or non-existent. Failure to do so will perpetuate the dogma that all fevers in all communities are due to malaria, which may be good for private sector market sales but poor for public health practice.

## Conclusion

The study could not demonstrate any evidence of malaria in Korogocho, an urban slum in the centre of Nairobi; however, fever was a common complaint and often treated as malaria with anti-malarial drugs. Strategies to reduce the population exposure to anti-malarial drugs should be a priority for all clinical services and drug vendors among the poor communities that make up Nairobi's informal settlements.

## Conflict of interests

The authors declare that they have no competing interests.

## Authors' contributions

YY designed and implemented the study. He performed the statistical analysis and drafted the manuscript; NM participated in the design and writing of the manuscript. RN took part in statistical analysis and writing of the manuscript; SO participated in writing the manuscript; RWS contributed to the study design, statistical analysis and drafting of the manuscript. All authors read and approved the final manuscript.

## Supplementary Material

Additional file 1 Treatment of self-reported episode by participants. Click here for file
