# Supplementary material for: Fever treatment in the absence of malaria transmission in an urban informal settlement in Nairobi, Kenya
Source: Malar J. 2009 Jul 15;8:160. doi: 10.1186/1475-2875-8-160 (PMC2717114; doi:10.1186/1475-2875-8-160)
Supplement: Additional file 1 — Treatment of self-reported episode by participants. [file 1475-2875-8-160-S1.doc]

| **Additional file 1: Treatment of self-reported episode by participants** | | | | | | | | | | |
| --- | --- | --- | --- | --- | --- | --- | --- | --- | --- | --- |
| **Characteristics** | | | **Total (%)** | | | **0-4 years (%)** | | | **5-14 years (%)** | **15 + years (%)** |
| **Total number of episodes** | | | **170** | | | **28** | | | **19** | **123** |
| **Took medication for the fever** | | |  | | |  | | |  |  |
|  | | Yes | 88 (51.8) | | | 12 (42.9) | | | 11 (57.9) | 52 (42.3) |
|  | | No | 74 (43.5) | | | 15 (53.6) | | | 7 (36.8) | 65 (52.8) |
|  | | Missing | 8 (4.7) | | | 1 (3.6) | | | 1 (5.3) | 6 (4.9) |
| **Total number of episodes treated** | | | **88** | | | **12** | | | **11** | **65** |
| **Origin of the medicine** | | |  | | |  | | |  |  |
|  | | Bought from a drug shop | 25 (28.4) | | | 2 (16.7) | | | 5 (45.5) | 18 (27.7) |
|  | | Bought from a pharmacy | 15 (17) | | | 0 | | | 2 (18.2) | 13 (20) |
|  | | Bought/Given from the health centre | 39 (44.3) | | | 10 (83.3) | | | 4 (36.4) | 25 (38.5) |
|  | | Traditional healer | 3 (3.4) | | | 0 | | | 0 | 3 (4.6) |
|  | | Other (e.g. in home or from friend/relative) | 6 (6.8) | | | 0 | | | 0 | 6 (9.2) |
| **Type of anti-malaria medicine** | | |  | | |  | | |  |  |
|  | | Sulphadoxine and pyrimethamine (SP) | 31 (35.2) | | | 2 (16.7) | | | 4 (36.4) | 25 (38.5) |
|  | | Amodiaquine (AQ) | 16 (18.2) | | | 5 (41.7) | | | 2 (18.2) | 9 (13.8) |
|  | | Coartem (artemether-lumefantrine) | 4 (4.5) | | | 0 | | | 0 (0) | 4 (6.2) |
|  | | Others (Chloroquine, Quinine) | 2 (2.3) | | | 0 | | | 2 (18.2) | 0 |
|  | Herbs | | | 3 (3.4) | 0 | | | 0 | | 3 (4.6) |
|  | Don't know | | | 32 (36.4) | 5 (41.7) | | | 3 (27.3) | | 24 (36.9) |
| **Delay between onset of the fever and beginning of treatment** | | | | | | | |  | |  |
|  | Same day | | | 18 (20.5) | | | 1 (8.3) | 4 (36.4) | | 13 (20) |
|  | Next day | | | 12 (13.6) | | | 1 (8.3) | 2 (18.2) | | 9 (13.8) |
|  | Two days after | | | 12 (13.6) | | | 1 (8.3) | 2 (18.2) | | 9 (13.8) |
|  | Three or more days after | | | 46 (52.3) | | | 9 (75) | 3 (27.3) | | 34 (52.3) |
